# Strategies and policies deteriorate occupational health situation in India: A review based on social determinant framework

**DOI:** 10.4103/0019-5278.58913

**Published:** 2009-12

**Authors:** Asish Kumar Mandal

**Affiliations:** Department of Ergonomics, Regional Occupational Health Center (Eastern), Block DP, Sector V, Kolkata - 700 091, India

**Keywords:** India, labor, occupational health, occupational health policy

## Abstract

Overwhelming evidence shows that hazardous work, working conditions, and environment fail to maintain homeostasis results in death or severe disability. Up to the 1980s, governments did not pay major attention to occupational health in developing countries, including India. The Bhopal Gas Tragedy, in 1984, was the turning point in the history of health and safety in India. It was time for the government to think deeply and review the existing legislative measures, for the upliftment of the occupational health situation in India. However, all the services remain grossly underutilized because of inadequate strategies, policies, and the lack of a proper monitoring mechanism, for occupational workers. The present study reviews the fact that Inaction or Destruction of Demands, Use of Power, Appeal to the existing bias of the system, and Exportation and Flexibility of the workers are some of the main reasons for the alarming situation of the Occupational Health Policy (OHP) in India. The existing and traditional condition of the laborers before and after independence is also highlighted in this article. Finally the threats are identified and options are provided to improve the health conditions of the workers.

## INTRODUCTION

Health is multifactorial and helps people to live well, work well, and enjoy themselves.[1] It is influenced by both internal and external factors of society in which people live. In the early twentieth century it was found that nobody worried about their health, then why did this sudden drift take place. It is difficult to answer this, but perhaps the hazardous work, working conditions, and environment manifest themselves in injuries to the human body. In extreme cases this also results in death or severe disability. Historically, it is also a time when the concept of a right to health is emerging and different international organizations are continuously trying to set strategies and policies for the human society to acquire good health.[2–3] Literature has revealed that since the 1980s, the governments have not paid major attention to the concerns regarding occupational health in developing countries, because of inadequate strategies and policies for occupational workers.[4–8] From the National Health Report it has been found that until 1983 India had no formal health policy, and therefore, lacked the formation of a health program design and all the existing services remained grossly underutilized because of poor facilities, a lack of community involvement, and the lack of a proper monitoring mechanism.[9] A new National Health Policy was announced in 2002. However, this also failed in its mission, as the planning for public health systems was debilitated.[10]

To summarize, in most cases budget allocations are inadequate to support the occupational health activities set up by governments and enterprises, which tends to kill occupational health in its stage of implementation, as exemplified in many developing countries.[11–12] In order to deal with this problem, it is necessary to understand the concepts of occupational health in India, supported by the working conditions of the laborers in India. Hereby, it is also important to identify the existing and traditional conditions of the workers before and after independence. It is also necessary to understand why a developing country like India lacks significant occupational health policies. To accomplish this, data was collected mainly from documentary sources, especially publications on occupational health, and also scrutinized from direct observation using in-depth interviews.

The overall article is divided into three major sections. Concept of health-related problems associated with the workers, which is supported by epidemiological evidence. To understand the labor hierarchy in India workers are classified through social stratification in the next section. The threats and options related to the strategies and policies are reviewed and presented in the last section of the article, followed by the conclusion.

## OCCUPATIONAL HEALTH IN INDIA

The conventional thinking on health dismisses it as a largely personal issue, something that is primarily influenced by personal behavior, habits, lifestyles, and so on. However, a range of historical and scientific evidence shows that the health of the communities and population is decisively influenced by a broad level of personnel. For example, Bimal a laborer employed in a Jute Mill Industry earned Rs. 60 per day, well below the stipulated minimum wage, in the Malda district of West Bengal. However, when the company incurred losses, the management decided to declare a lock-out, and closed the company. Therefore, his family had no option but to work as ‘contract laborers’ in a Brick Company and to live in a poor unhygienic slum area for a long time. One day while carrying bricks, he slipped off the ramp, broke his spine, and died. There was no compensation for his family, and as a consequence his young child had to carry water for other laborers working in the brick company. However, the tragedy of the story was that unknown to the child or the consumers of the water, the water contained trace elements of arsenic. (Self-reported).

## EPIDEMIOLOGICAL EVIDENCE

The 1931, the Royal Commission found the state of working and living conditions of Indian labor to be ‘horrible’.[13] Almost about 40 years later, in 1969, the National Commission found that both working and living conditions had improved somewhat in some sectors, but not in all. The Commission made 300 conclusions and recommendations in general, but effective enforcement was lacking.[14] In India, traditional public health concerns such as malnutrition and reproductive health gets importance in the health policy. Industrialization and globalization are changing the occupational morbidity drastically. New pathologies such as cancer, stress, heart diseases, and so on, are on rise. These new transitions pose challenges to the healthcare system, with high costs of healthcare. At the same time a general awareness about occupational safety and occupational and environmental hazards are not spread to the society, which ultimately leads to disease.[13]

The World Health Organization estimates occupational health risks as the tenth leading cause of morbidity and mortality.[15] The burden of disease from selected occupational risk factors amounts to 1.5% of the global burden in terms of Disability Adjusted Life Years (DALY). The World Health Report 2002, has reported that occupational risk factors account for a number of morbid conditions globally, including 37% back pain, 16% hearing loss, 13% chronic obstructive lung disease, 11% asthma, 10% injuries, 9% cancer, and 2% leukemia.[15] According to Census 2001 and the Director General of Factory Advisory Services and Labor Institutes (DGFASLI) Report (1998), the current burden of accumulated occupational diseases in India is estimated to be at around 18 million cases.[16]

The interconnection between socioeconomic factors such as poverty, income, occupation, class, and so on, and health have a long history, which has been explained by many great philosophers, such as, Villerme, Engels, Chadwick, Mckeown's, Dubos, Marmot, and Wilkinson, in their own style. Despite proper evidence from epidemiological data or information systems, several pieces of information are available from to understand the occupational health situation in India. DGFASLI indicates that such linkages need to be contextualized within the developmental discourse globally.[17] Leigh *et al*., have estimated an annual incidence of occupational disease between 924,700 and 1,902,300; and 121,000 deaths in India.[18]

Work hazards are also responsible for occupational diseases, and this was first identified in 1948. The Indian Council of Medical Research reported that the prevalence rates of silicosis in Bihar mines is 34.1%,[19] of pneumoconiosis in coal mines is up to 45%[20], and of byssinosis in textile workers is 8.4%[21] in different parts of India. Similarly, for asbestosis, silicosis, and lead poisoning, the reported range is between 6.5 and 30%, 16 and 57%, and 9%, respectively. For carbon disulfide and manganese poisoning the reported rates are as high as 27% and 24%.[22]

Census 2001 reported that the growth percentage for female workers wais higher than that for male workers from 1991 to 2001. The proportion of the male: Female working population, which was 78:22 in 1991, changed to 68:32, in 2001. This increase rate led to certain concerns, such as, adverse effects on reproduction, exposure to toxic chemicals in the workplace, musculoskeletal disorders, because neither the tasks nor the equipment they used were adapted to their build and physiology. In addition, the female workers had specific stress-related disorders, resulting from job discrimination (such as lower salaries and less decision-making), a double burden of work (workplace and home), and sexual harassment.[23] Occupational and environmental concerns were not two different issues. For example, as many as 10 million industrial or mine workers in India could be exposed to asbestos or other dust at concentrations that could be of concern to their health.[24]

Therefore, to decisively improve the health of a large group of people; the right approach must be in the form of a movement that moves toward a specific goal that is conducive to health. Innovative interventions including work organization change, participatory training, cleaner production, control banding, national and international coalitions, and participatory approaches to improving the work environment are needed.[15]

## SOCIOLOGY OF WORKERS HEALTH IN INDIA

In India, with local medievalism and multinational modernism existing side by side, the traditional labor-oriented markets have changed toward more automation and mechanization. Here, depending upon who is employed to work where, how, and at what level of exposure to hazard labor — the history of India may be divided into three broad categories of permanent, temporary, and contract workers.[25] In this section the nature of the different strata of workers along with their determinants is discussed.

### Social stratification

The report of the Labor Investigation Committee in India revealed that here, in both industrial and agricultural sectors, caste composition and social hierarchy have been noted at the national level. As a result of this, most of the low-caste families and tribes, including Harijans, are engaged in low-level occupations, such as, coolies, earth movers, rickshaw pullers, and so on, and are mostly migrant laborers, coming from the states of Bihar, Uttar Pradesh, West Bengal, and Madhya Pradesh.[26–27]

In India, the power for recruitment is under the hand of intermediates of factory owners commonly known as ‘Sardars’ or ‘Mistrys’. These jobbers have the power to engage and dismiss workers and the practice of bribes is common for both employment and non-employment. False promises of high wages, housing, and short working hours, are common methods for attracting laborers.[28] A report of the Labor Investigation Committee, 1946, revealed that the impoverished working class, such as, the landless, unemployed, marginal tribals, and peasants who lost in competition to the growing industries were forcefully included to work in mines as well as plantations by British Colony in India, using allurement, deception, and naked violence.[29] However, another category of labor, namely the ‘contract labor,’ is the most oppressed among the deprived, and is found to have escaped most of the provisions of Labor Acts, especially the factories Act, the payment of Wages Act, the maternity Benefit Act, and so on.[30]

The study of Majumdar and Singh indicates that ‘unskilled labor’ mostly lived in unhygienic environments, resulting in a high rate of malnutrition and communicable diseases.[31] In this social hierarchy, another group, namely, women and children were well discriminated from the society. They were absorbed in both agricultural and non-agricultural sectors due to shortage of labor in peak requirement seasons, at almost one-third of the salary as compared to an adult male.[32]

Therefore, social inequality has serious implications, not only at the work places, but also in the house.[32] Labor from low socioeconomic families have to do unskilled jobs and jobs that no one else takes up because of the associated hazards related to poor environmental conditions, and therefore, become depressed and exposed to more illness.

### Profit and deterioration of health status among workers in India

Participation of the workforce in the industrial sector is on the rise since our independence in 1947. It was argued that the expansion of industries and job opportunities were the first demands of development, without which achievement of health becomes mere talk.[33] As, the concept of general awareness about occupational safety and occupational and environmental hazards were not spread forward in the society toward the poor working conditions, it resulted in the deteriorating health conditions of Indian labor. The driving force behind Indian industrial growth has been the desire for profit maximization, and Indian ‘socialism’ has merely supplemented and mystified this thrust.[34] Therefore, search for higher profits has not only led to the neglect of workers welfare, but in fact has created a situation wherein work hazards have increased and become more mortal.

### Lack of significant occupational health policies in India

In developing countries, governments mainly claim health-related policies that favor the workers' health needs. In this respect it is worth mentioning that the elitist[33,35] as well as the structuralist theories[19] are mainly responsible for the failure of significant occupational health policies in developing countries. In India inaction or destruction of demand and use of power appeal to the existing bias of the system,[36] and also exportation and the flexibility of labor structure may be the most probable reasons for poor occupational conditions around the nation.

## INACTION OR DESTRUCTION OF DEMANDS

Inaction or destruction of demand was found to be an effective reason for non-decision making, which assigns low priority to occupational health. Here, the powerful may not attend to, listen to or hear demands as articulated by the less powerful.[37] If such demands do get permission into the political agenda, they are suppressed in the processing and execution stage through bureaucratic and official delays, or by passing laws that are not implemented.[38] Three situations illustrate such inactions and destruction of demands:

1. Under funding and poor economy kills initiatives that propagate toward improved occupational health.
2. Role of higher authority and trade unions at work places.
3. Corruption among the workers regarding implementation of occupational, health-related programs.

### Under funding of occupational health programs

In developing countries like India, the legislation designed to deal with occupational health matters is broad and even profuse in many cases. However, sometimes it cannot be applicable even to basic matters. Some of the most obvious problems are the lack of national and international funding, human and technical resources, weak strategies and policies, and more reasons, thereby weakening the idea of implementing health and safety programs.[22,23] India has taken occupational health on board by enacting legislation and creating structures for its implementation. However, in most cases budget allocation is insufficient for the government and enterprises, to support occupational health activities.

The main reason for underfunding is lack of political will rather than lack of funds.[39] Study of Sekempi in Africa concluded that funding to start any health training institution or the need and availability of money for occupational health programs are purely political decisions, whether in the Government Cabinet or in the University Senate/Council.[40] Therefore, it can be concluded that in most of the third world countries, allotment of resources is not the problem, the problem is the release of resources for appropriate use.

### Role of higher authority and trade unions at work places

Lack of implementation, however, seems to be an effective agreement between trade unions, resulting in a formation of a new kind of conflict between workers and management in both the organized as well as the unorganized sectors. A laborer of a Cotton Industry in Ahmedabad reported to me that managers hold a dual role in the industry; they signed a negotiated agreement with trade unions obligating the companies to provide a personal protective device to every worker. However, this agreement remained on paper only, hence, the companies had to provide only a few workers with personal protective devices.

### Corruption among workers regarding the health programs in India

Corruption, greediness, and lack of knowledge of health and safety among the workers not only fails to bring about a successful escape from poverty, but also pushes both the informal and formal sectors of economy toward vulnerabilities. Governmental as well as non-governmental schemes are sometimes found to be ineffective due the above-mentioned reasons, which are reflected from the experience of my own home town that is Malda. National Thermal Power Corporation (NTPC), Malda distributed insulated shoes for the safety of laborers, but the tragedy of the fact is that the laborers themselves sell their own shoes to get money and demand for the next shoes. Such a situation reflects how corruption, carelessness, and biasness among the workers are responsible for the failure of occupational health policies in India.

## USE OF POWER

Sometimes demands also get stifled through exercise of power. Public and private officials generally use power to suffocate occupational health measures through mechanisms such as centralization and monopolization of the decision-making power and weakening of organized groups through cooptation.

### Centralization and monopolization of the decision making power

Monopolization of decision making at the activity level causes certain impacts on occupational health. First, it isolates workers' leaders from the decision-making over health and safety issues at workplaces.[41] Second, it instills fear among trade union leaders and the workers' representatives, preventing articulation of their demands.[42] Third, the centralized decision-making structure creates loopholes for neglecting health and safety needs and problems by not giving the public sector companies sufficient decision-making power over social expenditure.[42] For example, from the report of the National Commission of labor, 1969, it is interesting to identify that the Indian Government has ratified only 30 of the 128 conventions adopted by the International Labor Organization (ILO). Of these, 15 were ratified before independence and 15 after independence. The Government of India is not bothered to include the other conventions along with consulting boards, managers, and workers of public and private sector companies.

### Weakening of organized groups through cooptation strategies

Most of the developing countries and their trade unions are not independent organizations. Therefore, occupational health and safety demands are diffused by reducing the potential power of trade unions through cooptation strategies, as found in Latin America and Africa.[43] Similarly, in India, where nearly 92% of the workforce is from the unorganized sector, trade unions have been pushed to a corner where the top priority seems to be fighting for job security and decent wages. From all over the nation, most of the time, our daily newspapers are replete with two-column-inch stories about the struggles and politics between trade unions and top level management ensuing closing of industries and factories day by day. Therefore, this irreparable damage has thrust workers to crush toward death, as evidenced from the newspaper headings.

### Appeal to the existing bias of the system

Decision of non-making can also be evidenced through an appeal to the existing bias of the system.[38] The powerful may dismiss a demand for change on the grounds that if it gets articulated it violates an established rule or procedure, although it can be achieved through the insistence on the use of proper bureaucratic channels[44] and appeals for adherence to the existing rules and decentralizations of occupational health responsibilities.[44–45]

## EXPORTATION AND FLEXIBILITY OF WORKERS

Due to globalization, the world economy and the factors responsible for the economy take an interesting meaning all over the developing countries, including India. It was found that laborers working in the informal sector had been exported from developing countries to the industrially developed countries. The forceful shift of workers not only affected the living and working conditions of the workers, but it also might be the probable cause of the very precarious situation today. Progressive disappearance of the benefactor state, significant reduction of social budgets, minimum social policies with little effect to reduce poverty, fiscal savings, furious competitiveness, weakened unions, dispersed social resistance, and the feeling of obligation to give priority to employment or income over job characteristics and quality are some of the prevailing effects of globalization.

### Occupational health services system in India

**Figure 1:**
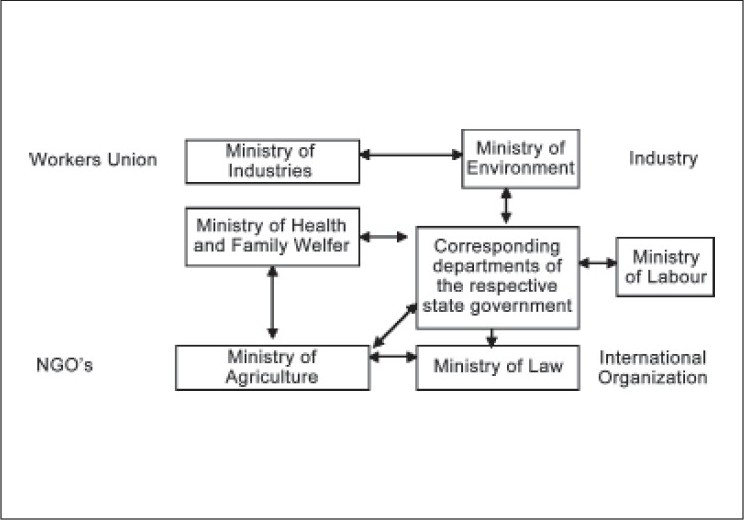
Organizations involved in occupational health and safety in India

In India the Occupational Health Services System operates at different levels and is linked to different levels of ministry and organization. It is diagrammatically represented in Figure 1. Here, it is also necessary to understand why State affairs continue, in spite of the remarkable advances that developing countries, particularly India, has made in industrial and agricultural production since the early part of this century?

To answer the question, the factors and socioeconomic variables are to be identified. The concept of non-decision making emerged through attempts to theorize power relations in the society. The three contrasting theoretical perspectives – pluralist, elitist, and structuralist features prominently explain power relation issues.

The pluralist perspective, rooted in the liberal democracy view, assumes the power to drive the policy process.[45] The elitist perspective sees the power as being concentrated in the hands of unrepresentative groups working in collaboration to confine the agenda and limit the area of public participation.[46–47] Thus, the main argument of the elitist theorists is that actors in the policy process have a capacity to keep issues that they control off the agenda.[48] The structuralist perspective appears most clearly in the Marxist theory. It observes economic imperatives as being paramount, limiting the scope for political intervention.[49]

## WELFARE SERVICES

In view of the plethora of deteriorating conditions in occupational health in India, certain welfare schemes and services were also present before independence. The Royal Commission noted that investment in health not only promoted the well-being of workers, but was also bound to produce a great economic advantage.[32] It covered a wide area from working hours to bonus for housing, health, and safety.

### Pre-independent India

Since 1929, there were 39 inspectors for eight provinces, with 8129 factories, whose main task was to report accidents and working conditions of laborers. From the Labor Investigation Committee Report, 1940, it was found that the incidence of accidents from that period was low.[32] Till the 1940s, public health services were largely provided by the provincial health services-but their structures were too weak to take care of the additional needs of industrial workers. The Labor Investigation Committee said, “Society as a whole must share the responsibilities for industrial development with all its attendant evils and to that extent must be regarded as liable to bear a part of the cost of medical facilities for hazardous and uncomfortable employment”.[34]

### Post independent India

Since independence, the national government initiated different schemes, acts, and research institutions. After independence some legal provisions for the protection of working groups are: The Factories Act (1948), the Plantation Labor Act, 1951, the Dock Workers (Safety, Health, and Welfare) Act, 1986, the Building and other Construction Workers (Regulation and the Employment and Conditions of Service) Act, 1996, the Beedi and Cigar Workers (Conditions of Employment) Act, 1966, Child labor (Prohibition and Regulation) Act, and the Insecticides Act, 1968. Legal provisions for the mining industry comprise of: The Mines Act 1952; 1955, Mines Rules; 1957, Coal mines regulation; 1961, Metalliferous mines regulation; and 1989, Oil mines regulation.[38] The Bhopal Gas Tragedy in 1984, was the turning point in the history of health and safety in India. It was time for the government to think deeply and review the existing legislative measures. A special chapter on occupational health and safety to safeguard workers employed in hazardous industries was added. In this chapter, pre-employment and periodic medical examinations and monitoring of the work environment are mandatory for industries defined as hazardous under the Act.[50] Strengthening of factory inspection, provision of housing facilities for different groups of working class, and implementation of laws and strategies for quality healthcare services are some of the major milestones. However, it was found that most of the welfare schemes were not enough to bring about a success story, as described by the labor investigation report. The provision of housing facilities for the working class had also failed due to poor observation, lack of infrastructure, allotment of money, and faithfulness among the hierarchical society.

## HEALTHCARE INSTITUTIONS AND ROLE OF NON-GOVERNMENTAL ORGANIZATIONS

The Adarkar Commission further concretized the welfare scheme by enacting the Employees State Insurance (ESI) act for the laborers, in 1948, after independence. The Act covered the insured workers and their families for sickness, maternity, employment injury, disability, and dependence.[38] The National Commission of Labor, in 1969, declared that one-third of the burden of medical costs for the worker must be taken from the employers for the employees. However, neither of the services provided adequate results and were found to be unsatisfactory. The major criticism of the ESI act was its poorly-staffed services/dispensaries, poor standard of clinics or hospitals, non-availability of medicines, and a total lack of attention for preventive services.[51] In spite of many legislative changes, the social relations between employers and employees remained same. Incidents and reports between 1884 and 1986 showed that things have remained unchanged after a century, even now child labor is exploited,[52] problems of bandaged labor remain unsolved, and protection of the health of the workers remains questionable. There are very few professional agencies like the National Institute of Occupational Health (NIOH) and its arm the Regional Occupational Health Center (ROHC), the National Occupational Health Center (NOHC), the Industrial Toxicology Research Center (ITRC), and the Central Labor Institute that are the institutes working for the betterment of work-related hazards since independence, in India. Perhaps this could be an important reason that a lot of non-governmental organizations (NGOs) are now interested in improving the health status of the workers. In addition to that, non-governmental organizations also play an important role, along with the governmental organization, for the upliftment of this alarming situation. The Indian Association of Occupational Health (IAOH) is India's leading NGO in Occupational and Environmental Health. Additionally, a number of NGOs and other groups have contributed to the strengthening of the health movement in India by means of specific forms of support. This includes research, which is relevant for taking up demands for campaigns, demonstration projects, and so on. While most such efforts are dependent on external resources, many have played a supportive role in strengthening the health movement. Instead of reform dictated by international capital, there is a need to press for reform from below, for reforming health services and drug policies radically, in a pro-people's direction.

## CONCLUSION

Improvement of occupational health requires strengthened organization and appropriate leadership in trade unions, conscious workers, who are able to control the work process, and generation of unbiased information about occupational health risks. Strategies and steps for the improved conditions of occupational health status include:

1. India urgently requires a modern occupational health safety (OHS) legislation with adequate enforcement, machinery, laws, occupational medicine, and a proper awareness program, to catch up with the rest of the world
2. Health awareness and factors to measure the safety analysis of the laborers working in particular industry
3. Empowering positive trade unions so that they can play a key role in demanding occupational health improvements
4. Making professionals available through training and development and enabling them to play an active role in the generation of information and knowledge through proper research
5. Work should be given depending upon two principles, which are, ‘worker fit for job’ and ‘fit the job for worker’, so that the employer generates interest on the work and avoids lack of attention
6. Need for policymakers to change their attitude toward occupational health and recognize that occupational health improvement is a vehicle for socioeconomic development
7. Impose a strict vigilance upon hazardous materials by investigators. The government should also weigh the pros and cons between environment and health costs of our people and cost of importing them from elsewhere
8. The labor report of West Bengal, India, records only minute details on hospital facilities and some data from ESI, with regard to the public health systems, which is not sufficient to understand the health and sociology of the workers. In India, however, there is no possible source from where we can get the actual report of allocation of money for the laborers. We have to depend on published reports and sources such as the Employment State Insurance (ESI), Workers Compensation Act (WCT), New Industrial Policy (NIP), Social Insurance, and so on, for allotment of money to occupational health, but only for organized and formal sectors, and not for the unorganized sectors. Therefore, it is necessary to record the life time job history, job titles, information on past occupations, industries, occupational conditions, and identification of other causal confounders. Record books for toxicity of chemicals, group of chemicals, industrial processes, and other complex mixtures along with their carcinogenic effects must also be kept. Issuing of job/occupation cards for the workers like ration card can be done for keeping records
9. Surveillance of diseases in industrial belts, maintenance of death certificates, and using record-linkage techniques between various resources may also potentially improve the research on occupational health
10. Quality assurance, creating awareness, accreditation, and capacity building (strengthening skills and developing competencies) will be needed in the field of occupational health. Model programs and pilot projects/surveys may be undertaken with the support from ILO/WHO and different NGOs like the Indian Association of Occupational Health (IAOH), for the unorganized sector.
